# Recent advances in engineering topography mediated antibacterial surfaces

**DOI:** 10.1039/c5nr04156b

**Published:** 2015-10-14

**Authors:** Jafar Hasan, Kaushik Chatterjee

**Affiliations:** a Department of Materials Engineering , Indian Institute of Science , Bangalore 560012 , India . Email: kchatterjee@materials.iisc.ernet.in ; Tel: +91-80-22933408

## Abstract

The tendency of bacterial cells to adhere and colonize a material surface leading to biofilm formation is a fundamental challenge underlying many different applications including microbial infections associated with biomedical devices and products. Although, bacterial attachment to surfaces has been extensively studied in the past, the effect of surface topography on bacteria–material interactions has received little attention until more recently. We review the recent progress in surface topography based approaches for engineering antibacterial surfaces. Biomimicry of antibacterial surfaces in nature is a popular strategy. Whereas earlier endeavors in the field aimed at minimizing cell attachment, more recent efforts have focused on developing bactericidal surfaces. However, not all such topography mediated bactericidal surfaces are necessarily cytocompatible thus underscoring the need for continued efforts for research in this area for developing antibacterial and yet cytocompatible surfaces for use in implantable biomedical applications. This mini-review provides a brief overview of the current strategies and challenges in the emerging field of topography mediated antibacterial surfaces.

## Abstract 2

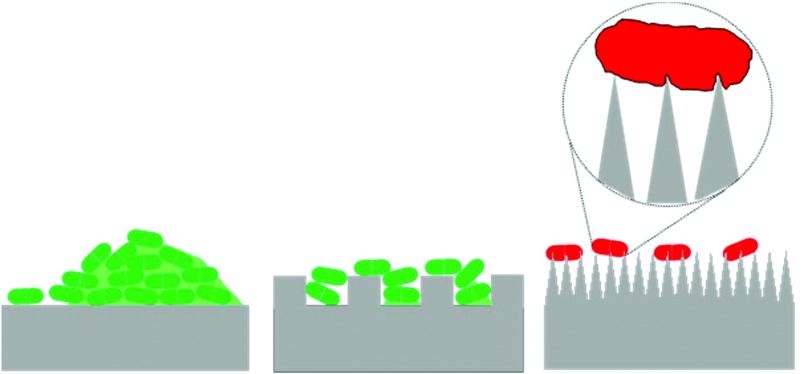
Recent advances in the field of topography driven antibacterial surfaces are presented. Micro-structured antibiofouling and nano-structured bactericidal surfaces are reviewed.

## Introduction

In an era of inter-disciplinary research where chemistry meets electronics and materials science is studied down at the level of nanoscale, scientists have now turned their attention to designing surfaces with remarkable functionalities such as antifouling, self-cleaning, anti-wetting, anti-icing, drag reduction, anti-reflective and self-healing.^1,2^ Antibacterial surfaces are critical in a wide variety of applications ranging from medical devices and water filtration to ship building and food packaging. Innovations in designing antibacterial surfaces have traditionally leveraged chemical and biochemical routes of surface modification to either minimize bacterial attachment to the surface or reduce viability of the adhered cells, and occasionally a combination of the above two strategies. Limited scientific work has been reported on the study of topography based approaches to develop antibacterial surfaces. This is perhaps because the field of microbiology has still a long way to go to fully leverage the recent advances in technology in physics and materials science, and *vice versa*.^3^ There is growing evidence in recent years that micro/nano-scale modification of material surfaces offers novel routes for designing antibacterial surfaces. Thus, this mini-review aims to highlight recent advances wherein bacterial interactions with geometrically modified surfaces have been studied. The review will also focus on the configuration of bacterial attachment on micro/nano-patterned substrates for biomedical applications, which typically place the most stringent demands among other applications.

### Bacteria–material interactions

**Fig. 1:**
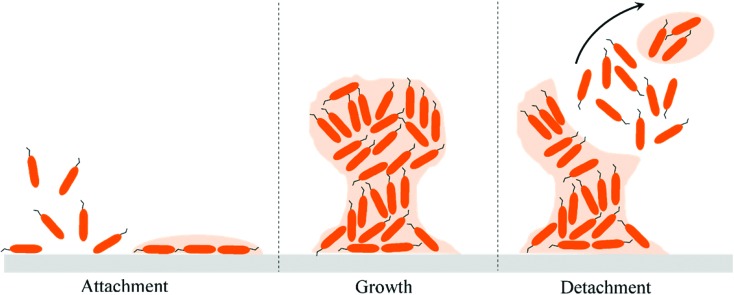
A schematic representing the typical stages of biofilm formation. The bacterial cells attach on the surface and secrete the EPS or slime layer to induce irreversible attachment. Thereafter, the biofilm often grows in the shape of a mushroom and finally few cells break-off to settle in another area of substrate to form a biofilm.

The first study of bacterial attachment on a solid substrate was reported in 1935 by Zobell and Allen.^4^ Since then the bacterial attachment studies have been extensively performed on a wide variety of natural and man-made substrates.^5–9^ A bacterial cell attached on a substrate is functionally distinct from a planktonic cell in the liquid milieu.^3^ Initial bacterial attachment to a surface is mediated by forces such as electrostatic and hydrophobic interactions, steric hindrances, van der Waals forces, temperatures, and hydrodynamic forces.^10^ The secondary attachment is then induced by bacteria-producing specific adhesins that bind with the substrate and/or by specific receptor ligands located on pili, fimbriae, and fibrillae of the cell. The adhesions and the slime layer, also called the extra cellular polymeric substances (EPS) produced by bacteria, assist in irreversible attachment and biofilm growth on the substrate.^11^ Biofilms are aggregates of bacterial cells attached on a substrate and the cells are encapsulated within the EPS. There may be several stages that lead to biofilm formation, of which the three main events presented schematically in Fig. 1 are as follows: (i) attachment events, (ii) growth and (iii) detachment events. Biofilms can form mushroom-like structures which cause infections and severe contamination on surfaces.^12,13^ Bacterial cells in the biofilms are less susceptible to the action of antibiotics and are of particular concern in the field of biomedical devices.^14,15^ Despite rigorous efforts to combat biofilm-associated infections, superbugs or multidrug resistant bacteria have started to emerge and pose a serious healthcare challenge.^16^ Yet few new antibiotics are being discovered. It took almost 30 years to discover the new antibiotic, Teixobactin.^17^ Therefore, novel antibacterial strategies are critical to address the emerging challenges related to bacterial attachment, growth and proliferation leading to infections.

Despite the vast literature on the interactions of bacteria with material surfaces, only a handful of reports have investigated the effect of surface topography on bacterial adhesion. Otherwise, much of the literature in this field has focused on the intrinsic chemical behavior of the surface that retards the attachment and growth of bacterial cells. The antibacterial surfaces are of mainly two kinds, (i) anti-biofouling or bacteria-resistant surfaces and (ii) bactericidal or bacteria-killing surfaces. For information on antibacterial surfaces based on chemical functionalities, readers are directed to some wide-ranging recent literature.^18–21^

### Surface topography: more than a geographical barrier

Topographical features of length scales comparable to bacterial dimensions, that is, micro- to nano-meter scales, offer a promising new approach to control cell–surface interactions. For decades, it was thought that surface topography can only resist bacterial attachment and in due course many antibiofouling surfaces were successfully fabricated.^22^ Many such studies have attempted to mimic the different unique surfaces found in nature.^23,24^ There are several properties exhibited by natural surfaces that have inspired researchers to mimic the surface design. Superhydrophobicity is one such unique property which is widely present in many animal skins, plants and insect wing surfaces among others and is attributed to the presence of hierarchical or non-hierarchical micro/nano-structured surfaces.^25^ Similarly, self-cleaning and antibiofouling structured materials are widely found in nature that make the surface void of dust, dirt and microorganisms. These kinds of surfaces include the shark skin, lotus leaves, rice leaves and many insect wings that keep biofouling under control.^26,27^ Besides these properties, optical properties such as antireflection and iridescence have also been observed in photonic nanostructures present in moth eyes, bird wings, insect wings and beetle cuticle.^28–30^

**Fig. 2:**
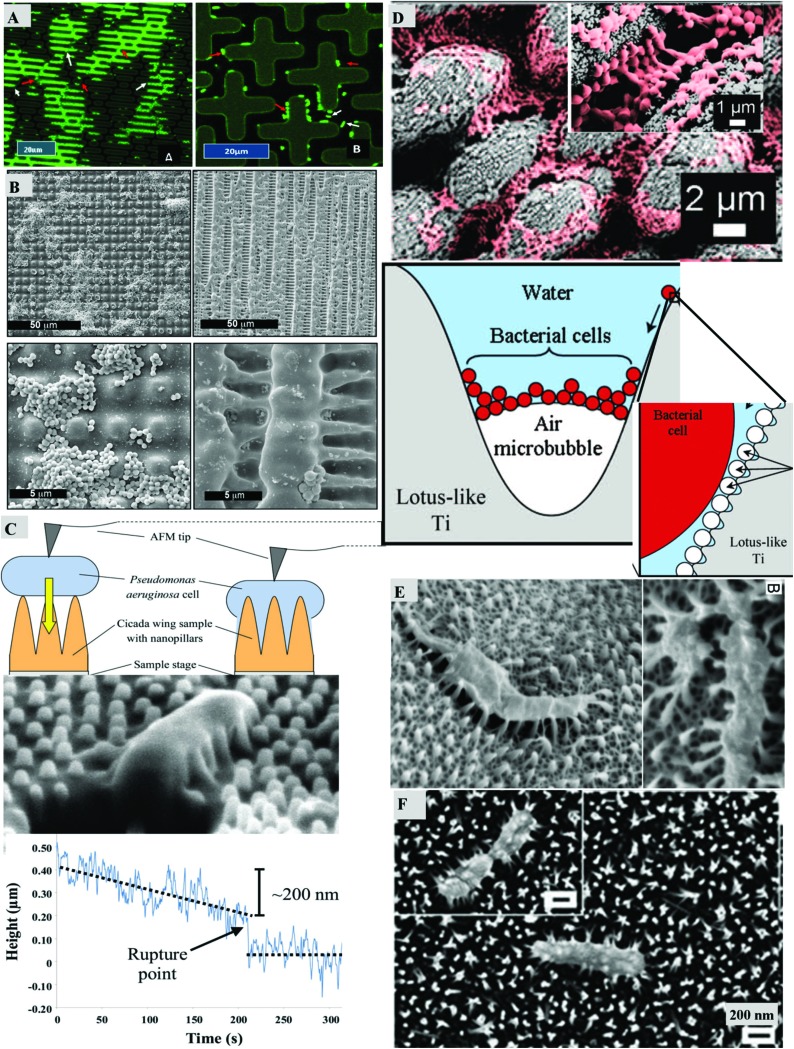
Bacterial attachment on micron and sub-micron patterned surfaces. (A) Bacteria are attached on the micropatterned surface which mimics the riblet-like patterns of the shark skin (left) and the cross-patterns (right), reproduced with permission from ref. 34. (B) *S. aureus* cells adhered on microstructured pillar and lamella structures, reproduced with permission from ref. 33. (C) The bactericidal activity of the cicada wing nanopillars is depicted by the AFM measurement of the sinking of the cell with height *vs.* time curve, reproduced with permission from ref. 42. (D) Coccoid shaped cells hang with air bubbles between the micropillar regions on the titanium surface which mimics the lotus like pattern, reproduced with permission from ref. 23. (E) Antibacterial activity of the gecko skin, reproduced with permission from ref. 24. (F) The ruptured morphology of *P. aeruginosa* cell is visible on the black silicon surface which renders the cell dead, reproduced with permission from ref. 43.

The summary of the recent literature on bacterial attachment on patterned surfaces is presented in Table 1 with some representative results compiled in Fig. 2. In most of the studies it has been shown that sub-micron and micro-sized features can minimize bacterial attachment although the topography does not have any direct influence on the viability of the adhered cells. In these studies, it is typically demonstrated that the bacterial cells prefer to settle in the regions between the micron sized pillars and more cells attach on the smoother surfaces than those attached on the patterned surfaces.^31–35^ These kinds of antibiofouling patterned surfaces were first explored on shark skin and lotus leaves and due acknowledgment must be given to these early discoveries.^22,36^ In a recent report, shark-inspired micro-patterns on a polyurethane catheter were shown to result in reduced bacterial colonization of the surface.^37^

**Table 1:** Antibiofouling patterned surfaces reported recently

Bacteria and their shape	Incubation time	Surface type	Surface features	Height	Width	Spacing	Observation
*Staphylococcus aureus*	Incubated for 1–4 hours	(a) Polystyrene, (b) polystyrene-*b*-poly(acrylic acid) (PS-*b*-PAA), (c) polystyrene-*b*-poly(l-glutamic acid) (PS-*b*-PGA) and (d) polystyrene-*b*-poly[poly(ethylene glycol)methyl ether methacrylate] (PS-*b*-PEGMA)	Open boxes	200	20	5	Bacterial immobilization is favored by a PAA block copolymer. Different polymer blends provide insight into bacterial isolation and positioning.^38^
Spherical			Square shaped	120	7.5, 19, 37	5, 6, 25	
			Crosshatched	140	20	—	
			Pillars	175	5	20	
			Lines	—	22	40	
			Hexagon	— (nm)	41(μm)	26(μm)	
*Staphylococcus aureus*	Incubated for 0.5, 5.5 and 24 hours	PEG microgel and silanized glass slide	Circular pillars	90	*α* = 1, 2, 3, 5	*β* = *α*/2, *α*, 2*α*	Attachment of an order of magnitude less than on the control; suggests diameter of 2–5 μm and spacing of 1–2 times of the diameter for optimum biofilm inhibition and promoting tissue growth.^35^
Spherical				(nm)	(μm)	(μm)	
*Enterobacter cloacae*	Incubated for 48 hours	PDMS	Cross pillars	23, 9	21, 4	5, 2	Bacterial cells attached on the walls and recessed regions between the patterns. Confirmed less attachment than the smooth PDMS control surface due to the less area fraction on patterned surfaces.^34^
Rod shaped			Hexagonal pillars	11	3	2	
			Hexagonal pits	7	3	5	
			Sinusoidal Sharklet™	3 (μm)	4, 8, 2, 16 (μm)	2 (μm)	
*Escherichia coli*	Incubated for 12 hours	Spinach leaves, PDMS and AGAR	Spatial symmetry of a natural surface	—	—	—	Bacterial cells aggregated in the valleys of the random topographical surfaces even after biocide treatment.^31^
Rod shaped							
*Escherichia coli*	Tested under real-time flow conditions	PDMS	Wells	5(μm)	10(μm)	7(μm)	Dynamic stability of the bacterial cells depends on the surface topography and flow parameters. The cells swimming on patterned substrates experience a differential and complex environment.^39^
Rod shaped							
*Staphylococcus aureus*	Incubated for 2 and 6 hours	Polystyrene	Line-like	1.6	1, 3, 5	—	In line- and pillar-like surfaces, spatial period of 1 μm had greater degree of bacterial attachment than on spatial periods of 5 μm. Although, cells on lamella-like patterns were significantly reduced compared to smooth control surfaces.^33^
Spherical			Pillar-like	1.8	1, 3, 5		
			Complex lamella	0.471, 4.3(μm)	2, 5(μm)		
*Staphylococcus aureus* and *Escherichia coli*	Incubated for 12 and 24 hours	Silicon wafer	Circular and square pillars	3(μm)	0.6, 0.8, 1, 1.2, 1.4, 2, 5, 10, 20(μm)	0.6, 0.8, 1, 1.2, 1.4, 2, 5, 10, 20(μm)	The microtopography patterned surface with equal width and spacing caused bacterial retention in comparison with smooth controls. *E. coli* adhered more on the 1.4–2 μm patterned surface while *S. aureus* adhered more on smooth controls.^32^
Spherical and rod shaped							

In addition to biomimetic surfaces, patterned surfaces with micron and sub-micron features have also been tested against bacterial attachment.^31–35,38,39^ On such patterned substrates, the bacteria are enclosed and surrounded by walls, wells, slopes, slants or other geometric curves. The confined surface structures with pillars of defined geometric shapes limit the attachment such that bacteria have less contact area between the pillars on the surface when compared with the smooth control substrates. Xu and Siedlecki reported that patterned arrays of micron and sub-micron sized ordered pillars on polyurethane significantly reduced the attachment and subsequent biofilm formation of *S. epidermidis* in buffer and blood plasma under shear flow.^40^ Interestingly, in a subsequent study they demonstrated that the wettability of the surface also affects the bacterial adhesion.^41^ When the polymer surfaces were made hydrophilic by plasma treatment, they noted that the sub-micron patterned surfaces but not the micro-patterned surfaces reduced bacterial attachment.

With the recent discovery of bactericidal insect wings exhibiting a nanostructured array of pillars,^42^ the study of surface topography mediated bactericidal surfaces has undergone a paradigm shift by redefining the old notion about topography based antibacterial surfaces. A number of reports have now focused their attention to designing and producing such topography based surfaces that actually kill the bacteria and not merely resist their attachment, which was earlier thought possible only through chemical routes. Thus, there is rapidly growing interest in fabricating anti-bacterial surfaces with nanoscale topography. Few studies on nano-structured bactericidal surfaces have been reported recently.^43–45^ Techniques such as deep reactive ion etching (DRIE) and nanoimprint lithography (NIL) have emerged as potent means for fabrication of such high aspect ratio nanostructured bactericidal surfaces.^43–45^ Black silicon consisting of anisotropic nano-structured pillars prepared by DRIE was the first study to mimic the topography of insect wings on a silicon surface that exhibited a bactericidal activity.^43^ We have recently engineered a “super surface” that exhibits superhydrophobic and self-cleaning properties in addition to bactericidal properties inspired by the insect wings.^44^ The DRIE technique which was used to produce the “super surface”^44^ was similar to the one which was used to fabricate the bactericidal black silicon^43^ but with slight variations in the processing parameters. The geometry and the architecture of the nanopillars of the “super surface” were rather different, as such the pillars were taller from the previously studied bactericidal insect wings and black silicon surfaces. The differences in nanopillar geometry can alter the killing mechanism and require further investigation for comprehensive understanding. Despite effectively killing the bacteria attached on the nanostructured “super surface” produced by DRIE, the surface was not compatible with eukaryotic cells thereby rendering it unsuitable for use on biomedical implants.^44^ Nevertheless the self-cleaning surfaces offer great potential to be used in surgical instruments, lenses and biosensors.

In another study, the patterned gecko skin was observed to be superhydrophobic, self-cleaning, antibacterial and yet cytocompatible.^24^ The tiny hairs (spinules) present on the gecko skin were able to lyse the bacterial cells incubated up to a period of 7 days. Nevertheless, it was shown that such gecko hairs are compatible with human dental pulp stem cells. The reason for the compatibility of the eukaryotic cells in this case is likely due to the presence of softer gecko hairs in contrast to our study. Although the stiffness was not reported in either of the two studies, the reported literature values of elastic modulus of the structured silicon surfaces(≈100 GPa) are greater by 6 orders of magnitude when compared to the elastic modulus of the gecko setal arrays (≈100 kPa).^46–48^ The elastic modulus of the substrate affects many cell functions such as morphology, migration, polarization, mechanotransduction, differentiation, regeneration, contractile forces and cell–cell signaling.^49,50^ In addition, the stiffness and geometry of the nanostructures will affect the ability of these pillars to mechanically rupture the cell membrane and thereby affect the target application of the material surface.^51^ For example, the surface with sharp nanopillars and high elastic moduli can be used for biomedical devices such as surgical instruments but may not be ideal for use as surfaces of implants where integration with the surrounding tissue is desired for the success of the device.

Plasma generated nanostructures have drawn significant attention for the modification of medical implants and instruments.^52,53^ In a recent study, plasma treated commercial sutures were etched for different time intervals to induce topographical changes and then tested for antibacterial activity.^54^ The plasma treatment induced a nanostructured lamellar pattern on the suture surface and upon bacterial attachment, only absorbable suture surfaces exhibited an antibiofouling effect where a one fold reduction in the attachment of *E. coli* cells was observed post 20 minutes of oxygen plasma exposure. Though the bacterial cells were not killed on contact with the sutures, however the low-cost plasma treatment offers a simple strategy for the fabrication and modification of nanostructured biocompatible and antibacterial surfaces.

### Synergistic effect of chemistry and topography

Some studies have utilized topography mediated bactericidal effects in combination with chemical groups to inhibit biofilm formation.^55–58^ Working on polymer nanocomposites, our group showed that carbonaceous nanoparticles such as graphene and carbon nanotubes inhibit biofilm formation by mechanically rupturing bacterial cells. Chemical functionalization of the nanoparticles with amine groups which are known to lyse bacterial cells further enhanced the bactericidal activity of the composites.^56,59^

In a recent intriguing study, diblock copolymers of poly(sulfobetaine methacrylate) (poly(SBMA)) and poly(propylene oxide) (PPO) were adsorbed on three kinds of surfaces consisting of smooth, convex and indented surface topographies.^60^ The self-assembled zwitterionic moieties exhibited considerable bactericidal properties as expected but the interesting observation was that on curved and indented surfaces, the differential orientation of the similar diblock copolymers increased the antibiofouling performance by limiting the interactions of the bacterial cells with these surfaces. The indented surface showed better antibiofouling ability than the curved surface as the bacterial cells were unable to adhere between the indents.

In another statistical study, multifractal analysis was utilized to define the attachment pattern of *S. aureus* and *S. epidermidis* cells on substrates of stainless steel and various bactericidal ceramic based (Ti–ZrN/Ag) silver coatings on the substrates of stainless steel.^61^ It was found that the clustering of *S. aureus* cells was more influenced by the chemistry on ceramic substrates and that of *S. epidermidis* cells was more influenced by topography on silver substrates. Similar comprehensive reports on the combinatorial effect of surface chemistry and topography using rigorous quantification of cellular behavior in differentially confined locations by statistical and mathematical analyses are needed.

### Stimuli-responsive antibacterial surfaces

Stimuli responsive polymers have been extensively studied in the past for their antibacterial activity, biocompatibility and other biomedical applications.^62,63^ A thermally responsive polymer, poly(*N*-isopropylacrylamide) (PNIPAM), is known to resist bacterial growth due to the differential polymer conformations above and below its lower critical solution temperature (32 °C).^64,65^ PNIPAM is often used as a carrier functionalized with an antibacterial agent that makes the surface antibacterial.^63,66–68^ Any direct effect of surface topography of the PNIPAM surface on bacterial activity has not been confirmed.

Aizenberg and colleagues have produced polymer based nano/microstructured surfaces that swell and contract based on the change in pH.^69,70^ Such pH responsive polymers can be used to tune the topography and thus produce antifouling surfaces. Other stimuli responsive antibacterial surfaces have also been actively studied in the recent past such as the renewable sacrificial skin based on the pilot whale, the self-assembly of peptide sequences and self-replenishing oil infused surfaces.^71–73^

Recently, topographical changes triggered by external physical stimuli have also been explored as a plausible strategy to affect the bacterial retention. The surface area and topography of the elastomeric silicone surface were changed in response to two kinds of external stimuli, namely voltage-induced and stretch-induced.^74^ The surface topography of the elastomer was changed from a smooth surface in the absence of an electric field to a surface with a crater-like pattern when an electric field was applied. It was observed that during the voltage-induced deformation of polymer surfaces, almost 95% of the adherent *Cobetia marina* biofilms were removed. During the applied voltage deformation, the effect of surface deformation in order to remove the biofilms was more dominant than the actual applied voltage itself. Also, the primary effect of surface deformation by stretching the elastomer uniaxially was studied and it was found that more than 80% of the *Cobetia marina* and *E. coli* biofilms were removed. In a more recent similar study, a hydraulic and pneumatic induced elastomeric surface released 90% of *Proteus mirabilis* biofilms from the strained surface.^75^ These kinds of biofilm debonding studies suggest a good application platform for biomedical implants along with some other biofilm removal mechanisms that may be triggered on demand.

### Summary and future perspectives

Novel approaches for designing an antibacterial surface mediated by topographical features are receiving increasing attention apart from the conventional chemistry based strategies. Although there have been some interesting recent developments in this field, more breakthroughs are essential to fully leverage the potential of current techniques to fabricate such structures for engineering materials used in preparing biomedical devices. Many of the currently reported techniques utilize micro- and nano-fabrication techniques which are essentially optimized for silicon wafers in the electronics industry. These techniques have to be adapted or novel techniques must be developed to impart antibacterial activity to wound dressing products, medical implants, surgical instruments, *etc*. that are typically prepared from degradable and non-degradable plastics and textiles, ceramics and metals and alloys, *etc*. In addition, such topography-mediated antibacterial surfaces on engineering materials will find use in other applications such as food packaging, industrial vessels and pipelines, ship building, *etc*.

Herein we have reviewed recent efforts on developing micron and sub-micron sized pillars to inhibit bacterial attachment in comparison with the smooth surfaces but such pillar topography is ineffective in totally controlling the bacterial attachment as cells find sufficient interpillar regions for attachment. Further optimization of the spacing to minimize attachment in the interpillar gaps could be utilized to design superior antibiofouling surfaces. The nanoscale spacing in the patterns of the cicada and dragonfly wings could serve as inspiration. Optimization of current methods and development of new fabrication techniques must be complemented with mathematical modeling and statistical approaches. It is important to recognize that the mere presence of nanoscale topographical features may yield an antibiofouling surface but not always a bactericidal surface.^76,77^ Though recent biomimetic approaches have yielded bactericidal surfaces exhibiting sharp nanopillars that can lyse the bacterial cells, not all such surfaces are cytocompatible. For use in biomedical implants, further optimization of the nanopillars is required such that the surface promotes the attachment and growth of mammalian cells. Perhaps softer polymeric pillars that rupture the small bacteria but remain conducive to much larger mammalian cells could offer a plausible solution. Further work is warranted to engineer such surfaces through development of novel micro- and nano-scale techniques.
